# Fiscal Success During COVID-19 Says Believe the Good News

**DOI:** 10.1007/s10272-021-0980-y

**Published:** 2021-08-05

**Authors:** Adam S. Posen

**Affiliations:** Peterson Institute for International Economics, 1750 Massachusetts Avenue, NW, 20036-1903 Washington, DC, USA

## Abstract

Too much blood in terms of unemployment and sweat in terms of intellectual effort has been spent on trying to determine the amount of fiscal space that economies have — our policy focus instead should be on what to do with the fiscal space that almost all advanced economies (and a surprising number of emerging market economies) actually have.

This realization begins with the failings of making misleadingly precise guesses about the output gap and various other not directly observable measures as a driver of policies within the state. Starting with Posen (1998, 2001) on restoring Japan's economic growth, there has been a mounting attack on official sector output gaps being biased downwards by repeatedly estimating recession outcomes as the trend. Also, building on this analysis of Japan in the 1990s versus 2000s, and subsequent experience globally, has been opposition to the idea that fiscal austerity or even just fiscal inaction are the right responses to a recession.

* The author thanks Madi Sarsenbayev for research assistance, and Olivier Blanchard, Karen Dynan, Jason Furman, Joseph Gagnon, Jacob Kirkegaard, Maurice Obstfeld, Peter Orszag and Angel Ubide for discussions of these issues.

## The fiscal response to COVID-19

The shift from the fiscal passivity and premature austerity of 2008-12 to the aggressive fiscal response to COVID-19 in 2020-21 has therefore been heartwarming as well as beneficial. As Furman and Summers (2020) pointed out, the fiscal response which took months in the US and a couple of years in the euro area in the global financial crisis, was far exceeded in size in both places in spring 2020 in a matter of weeks. At least as importantly, both the US and euro area governments have committed to avoiding the devastating fiscal reversals they pursued – and advocated for others – in mid-2010.

Perhaps some of this shift in fiscal response is due to greater perceived universality of the pandemic, and it being deemed a supply shock rather than the fault of financial moral hazard. That would be unfortunate because distinguishing the shocks rather than seeing the first response as a mistake would be the wrong conclusion.

Fiscal activism in response to COVID-19 succeeded along every dimension, and it did so in ways that indicate it would also have been successful in response to the global financial crisis (or previously to Japan's Great Recession). Response was rapid and commensurate in scale, with some targeting and some built-in sunsets – it was not too late and persistent, as often assumed to be inevitable. Multipliers were high and lags were few. Fiscal-monetary cooperation worked globally to allow for simultaneous large-scale bond issuance, with markets accepting the response. Exchange rates and inflation expectations remained stable. Public investment (in vaccines and medical provision) crowded in private investment.

Even most emerging markets and some low-income economies had macroeconomic policy space to run countercyclical macroeconomic policies after April 2020, not as much as for the advanced economies, but enough that capital did not fly out of their bonds. In fact, public sector issuance by some emerging markets and lower middle-income economies continued to increase (Bogdanova et al., 2021). So fiscal room is more the global rule than the exception, though Mauro and Zhou (2021) and Blanchard et al. (2021) offer some reasons for caution.

We need to overcome the widespread bias against believing in fiscal good news. Finance ministry officials are institutionally encouraged to be skeptical, to see their role as conservatives, to say no to spending proposals. Whether trained as lawyers or economists, they are schooled in looking for clever counter-intuitive effects of well-intentioned policies. At some point, however, they and we have to trust the accumulation of evidence in favor of fiscal space being larger, and fiscal policy being more constructive, than these pre-conceived notions allow.

The pandemic stress tested longer-term patterns of economic response to fiscal policy which already had emerged in light of the low-interest rate trends. When public expenditure is put to good use, both markets and citizens can appreciate it. The savings glut/shortage of safe assets has persisted and shows no sign of going away even as debt-to-GDP levels have risen. The arguments for going the other way – expansionary austerity, crowding out of private investment, the relative importance of long-term goals over stabilization against shocks, fiscal discipline inducing structural reform – have been contradicted by experience, most prominently and repeatedly in the euro area (Posen, 2005; Kirkegaard and Posen, 2018; Blanchard et al., 2021).

## EU – US comparison

Getting more specific about new EU-US comparison, though, requires looking at how the respective governments used their fiscal room. The fundamental point is that the US was playing catch-up with the EU in dealing with social dislocation. Compared to almost every EU member economy, the US does not have a sufficient welfare state and does not have a large set of automatic stabilizers, which is tied to the US having a paltry public sector. As a result, a lot of the policies that Washington enacted in 2020-21 were ad hoc, making up for these failings.

Relatedly, there is a lot of creative research work to be done to disentangle the right approach to dealing with short sharp jumps in labor dislocation. In much of Europe, the emphasis was on furloughs and kurzarbeit, relief that was mainly disbursed to maintain the employee/employer relationship; in contrast, as many have pointed out, in the US, emphasis was on layoffs and separation from employers in order to get unemployment insurance.

In the short run, Europe was probably wise (or fortunate) to go on the route that it did and not create additional frictional unemployment. Even if extent of firm- and job-specific human capital is often exaggerated, the more the post-pandemic economy looks like the pre-pandemic economy in terms of geographic and sectoral allocation, the less the costs of encouraging workers to stay tied to their jobs. If the replacement of workers by capital (automation) is in part path-dependent, not a question of obvious optimality or progress, then encouraging fewer mass separations may also pay off for longer-term welfare at little cost. As the US converges back to its old structures, with some one-time jump in automation, this conjecture will be increasingly borne out.

That said, both the US and Europe remain problematic on the fiscal design side in some common ways. First, the scale of transfers across jurisdictions within the federation in both entities remains too low. While it is obviously very different to speak of the federal response in Europe, and there has been promising progress of mutual fiscal policy just this year, including some issuance of euro-level bonds, at the level of the monetary zone, insufficient transfers persist between regions. While the US has more inter-state transfers within the union, swings in state and local government revenue – with balanced budget requirements – significantly offset national countercyclical response.

The second point that remains an issue is the poor design of automatic stabilizers, or rather the emergence of automatic stabilizers as the mere residual of other tax and spending decisions. This is not just about insufficient spending during downturns. There has to be some credible sense that you are going to pick up revenues in the later years, which is partly an institutional matter and partly a political matter. Either way, what we have in the US and

Europe is a failure to create a credible cyclical response in both directions. Rather than trying to guide them via the unobservable output gap, which usually includes insisting prematurely that the gap is about to be closed and that we need to immediately start collecting taxes, we should come up with a labor market-based cycle or some kind of lagging indicator cycle. One also might consider counter-cyclical taxes on real estate that also have financial stability benefits.

The third point is one of governance. This is of course even more different between the US and Europe, though both purport to rely on some amount of technocratic guidance and "scoring" of spending and deficits. One way to respond to this challenge is to come up with more sophisticated and flexible forms of debt sustainability analysis (e.g. Blanchard et al., 2021). Another is to try to refocus targets on sounder measures of fiscal room (e.g. Furman and Summers, 2020). But in the end, there is no institutional fix via fiscal rules for politics (Posen, 2005).

At present, European governance is at less risk than US governance: Due to Republican obstructionism, the US Congress has been unable to pass any long-term budget or to reliably raise taxes when needed. This is something more akin to Italy or Argentina in the 1970s-80s or is getting there following a breakdown of fiscal stability because of political fragmentation. The fiscal space is taken away not due to high quantity of debt, but due to low quality of ability to cope credibly with rises in debt. That is why Japan is able to bear such high-debt levels, and why we should worry that the US may not continue to be able to do so at a lower level.

### Too much public debt?

There are no simple limits, or even robust rules of thumb, for when an economy issues too much public debt. But current fears of reaching such numerical limits are excessively restrictive. As set out in Blanchard (2019, 2021), a good starting point is to look at when the safe rate of interest is below the rate of income growth. A lasting negative r-g differential implies that an economy can run a primary deficit in line with r-g and keep its debt-to-GDP ratio constant. As we are seeing right now in response to COVID-19, a persistent negative r-g differential also means that an economy can issue additional debt for a one-off (emergency) program, and never need to raise taxes to pay for it. Fiscal space is particularly valuable when the cost of recessions is high relative to trend growth, and monetary policy is near the effective lower bound for interest rates.

Of course, the government interest rate can jump for reasons of fundamentals or even self-fulfilling panics. But when the central bank supports the economy and the government, this is unlikely for advanced economies (Posen, 2010), as the 2012 "whatever-it-takes" moment demonstrated. There are many reasons to believe in continued low rates for the advanced economies (Posen, 2011), including:

- demographics and cross-country convergence increase global savings;
- worker bargaining power over wages remains limited;
- pace of technological progress remains slow at frontier;
- diminished risk appetite from private investors raises safe asset demand;
- repeated inflation undershoots anchor inflation expectations;
- rates in the US, and to a lesser degree in China and the euro area, lower the floor for others;
- stability of government regimes is high if not increasing;
- lower levels of taxes and foreign currency debt make consolidation feasible if necessary.

In any event, declining real rates are the continuation of a centuries-long downward trend (Schmelzing, 2020).

Still, as argued in Orszag et al. (2021), humility about forecasts for r* is justified, if one looks at the failings of previous official- and private-sector forecasts of long-term risk-free rates. What are the policy implications if we were to take uncertainty about interest rates seriously and humbly?

Orszag et al. (2021) propose equipping policymakers to face deep uncertainties about future interest rates as well as hard-to-predict global shocks (including climate risks). They reject fiscal anchors – simple limits on deficits or debt as a share of GDP – because any attempts to modify such targets for a given period will not see needed changes. Instead, they propose making the annual budget respond more automatically to economic swings, while making government programs respond more automatically to long-term fiscal pressures (embed adjustment mechanisms in health care and pension programs). Relatedly, investments like those for climate adaptation and mitigation should be part of a sustained counter-cyclical infrastructure investment program. In a low-rate environment, debt maturities may also be extended to hedge against interest rate changes. This set of recommended policies, though, is perfectly consistent with what is outlined here as a better use of fiscal capacity assuming low rates – the policy implications are still for greater fiscal activism and less attention to numerical debt limits or rules.

Finally, better use of fiscal room includes a plea that we should be doing bottom-up budgeting; that is, we need to decide what it is we need to spend on, including pandemic preparedness and climate change and so on, make the commitment, and then raise the taxes to meet it. Kirkegaard (2018) shows through historical fiscal episodes in the US that this is usually how it has gone in the US – that the government does something constructive, whether it is for infrastructure, war, or a welfare state, and then is able to find the money necessary for it. Arguably, that is what we have seen recently in the EU with mutual transfers taking place in response to the pandemic, and the specifics of the Union getting its own means to pay for it coming along later.

## Conclusions

What does the good news about fiscal policy really add up to? The COVID-19 fiscal policy response should not be viewed as something to emerge from, but rather as a model going forward in which stabilization plays just as important of a role as structural reform. The priority should be establishing a means to sustain and evaluate longer-term infrastructure (green) investment and to expand automatic stabilizers rather than spending more effort on fiscal rules and sustainability. To this end, monetary coordination can and should be an enabler for fiscal policy. Finally, it is important to remember that fiscal restraint is not its own reward.
